# Supplementary material for: Effects and predictors of intravenous lidocaine infusion for patients with fibromyalgia
Source: PLoS One. 2025 Jul 24;20(7):e0320463. doi: 10.1371/journal.pone.0320463 (PMC12289028; doi:10.1371/journal.pone.0320463)
Supplement: Attachment — Submitted filename: Response to editors and reviewers.docx [file pone.0320463.s005.docx]

Dear Dr. Gouda,

We are grateful to you and the reviewers for the constructive feedback and comments. We have addressed all the questions and comments raised.

**Responses to the editor:**

*1. Abstract: Briefly give the key findings of your study. Include key numeric data (including confidence intervals or p values).*

Key numeric data and p-values have been added to the Abstract.

*2. Patients and Methods. How were the patients selected (e.g., consecutively, randomly, or selectively)?*

The information on patient selection has been added to the Abstract and the Methods (1st paragraph on page 6).

*3. All studies must be reported according to the relevant Equator network guideline:* [*https://www.equator-network.org/*](https://scanmail.trustwave.com/?c=8248&d=17qo6P56iEd8Y8BQNf4bz7mevTYHE_bBRVkFKXmScw&u=https%3a%2f%2fwww%2eequator-network%2eorg%2f) *In your manuscript, you need to state in the Methods section that you have followed relevant Equator guidelines. For example, if you use STROBE: ‘The reporting of this study conforms to STROBE guidelines. (Insert new reference number).*

Please see the Abstract on page 2 “This study adheres to RECORD guidelines for reporting studies using observational routinely collected data. It was conducted retrospectively and employed data derived from clinical records of patients who received standard care.” and Methods on page 5 “We adhere to RECORD guidelines for reporting studies using observational routinely collected data [26].”

4. *More details should be provided in terms of the IRB approval.*

Please see Methods on page 5 “This was an NHS registry study using routinely collected patients' data, which was approved by the R&D Lead of Guy's and St Thomas’ Hospital, London.” There isn’t a specific IRB number available.

*5. Discussion: You concluded that IV lidocaine infusion showed higher effectiveness in female patients compared to male patients. A comparison of your results and the relevant previous studies showing gender differences in FM should be made. For example: DOI:10.2147/OARRR.S358255*

We have added some more discussion. However, it is difficult to compare our results directly with those published in previous studies, as the protocols of lidocaine infusion and the parameters of assessments were different from one study to another.

**Response to Reviewers**

**Reviewer #1:** *The references cited in the manuscript need revision. The author needs to ensure the PLOS ONE journal's guidelines. For proper formatting, I recommend the authors visit the journal's author guidelines.*

We have checked the format of our manuscript following the author’s guidelines closely and made the relevant changes where needed.

*The authors identified 50% pain reduction as a reference for responders. I recommend the authors briefly justify the relevance and clinical significance of the 50% pain reduction criterion in the setting of fibromyalgia treatment.*

Please see the Methods on page 8. “A clinically important difference is defined as a 30% pain reduction or a 2-point improvement on the NRS (0-10) [30]. We adopted a stringent 50% pain reduction to identify a group of patients that fell in the category of “very much improvement” and exceeded a 2-point pain reduction.”

*The manuscript did not reflect the characteristics of the control group. While there was mention of controls being matched for age, sex, etc., were there any other control group characteristics that could have had an effect on QST parameters?
It would be interesting if there is a brief description of control group characteristics*

We have compared the two groups: responders and non-responders, based on the responses to IV lidocaine infusion. QST parameters and phenotypes were similar in responders and non-responders. Responders were mostly females and significantly younger than non-responders. The raw data of QST was transformed to Z scores. Please see the Methods on page 7. “The results of QST were transformed to Z-scores (patient measurement-mean of age- and gender-matched healthy controls )/SD”

*Minor:
1. In the 2nd paragraph of Methods titled “Questionnaires, screening blood tests,
and clinical investigations”, there are no descriptions about the screening blood tests.*

Please see Methods on page 6. Screening blood tests, including full blood count, and renal and liver function tests, were performed.

*2. Due to the large number of abbreviations within the manuscript, it is advisable to have a table listing all of the abbreviations.*

We have added a list of abbreviations based on the suggestions. Please see the abbreviations on page 3.

*3. The authors set up the responder standard at 50% or greater pain reduction. According to the literature, the responder standards range from 20% to 50% pain reduction. It will be helpful to discuss how the responder standard was chosen to get clinically meaningful data.*

Please see the Methods on page 8. “A clinically important difference is defined as a 30% pain reduction or a 2-point improvement on the NRS (0-10) [30]. We adopted a stringent 50% pain reduction to identify a group of patients that fell in the category of “very much improvement” and exceeded a 2-point pain reduction.”

**Reviewer #2:**

We have made changes based on the comments made in the PDF file.

I hope the manuscript is in a satisfactory state for publication. Please inform me as soon as possible if further amendments are needed.

Regards,

Xuenong Bo
